# Near-infrared fluorescence-guided metastasectomy for hepatic gastrointestinal stromal tumor metastases using indocyanine green: A case report

**DOI:** 10.1016/j.ijscr.2020.12.058

**Published:** 2020-12-20

**Authors:** O.D. Bijlstra, F.B. Achterberg, Q.R.J.G. Tummers, J.S.D. Mieog, H.H. Hartgrink, A.L. Vahrmeijer

**Affiliations:** Department of Surgery, Leiden University Medical Center, Leiden, the Netherlands

**Keywords:** GIST, gastrointestinal stromal tumor, ICG, indocyanine green, NIRF, near-infrared fluorescence, FFPE, formalin fixed paraffin embedded, H&E, hematoxylin and eosin, HPF, high power field, Indocyanine green, Gastrointestinal stromal tumor, Near-infrared fluorescence-guided surgery, Liver metastases, Case report

## Abstract

**Introduction and importance:**

Gastrointestinal stromal tumors are the most prevalent mesenchymal tumors of the gastrointestinal tract. Distant metastases are most often found in the liver or peritoneum with surgery being the preferred treatment option. In our center, fluorescence-guided surgery with indocyanine green is used as standard-of-care for hepatic metastases in colorectal cancer. This case report describes fluorescence-guided metastasectomy for a hepatic gastrointestinal stromal tumor in two patients undergoing open liver resection and radiofrequency ablation.

**Case presentation:**

A 69-year old women was seen during follow-up after laparoscopic resection of a GIST in the lesser curvature of the stomach. Contrast-enhanced computed tomography imaging showed two suspicious lesions in liver segment VI and VIII. Intraoperative near-infrared fluorescence imaging of the liver clearly revealed the lesion in segment VIII, and an additional lesion in segment V – which was not seen on preoperative CT-imaging, neither on intraoperative ultrasonography. The lesion in segment VI was not seen with NIRF imaging due to its deeper location in the liver parenchyma. The second case is an 82-year old man who was also diagnosed with liver metastases from a GIST in the stomach and was scheduled for near-infrared fluorescence-guided liver resection and radio frequency ablation.

**Clinical discussion:**

In this case report we demonstrated the feasibility of fluorescence-guided surgery in detection of liver metastases and treatment planning of two patients with hepatic GIST metastases using indocyanine green.

**Conclusion:**

NIRF-imaging with ICG is useful for identification of preoperatively discovered lesions, surgical resection planning and margin evaluation, and for detection of additional hepatic GIST metastases.

## Highlights

• Near-infrared fluorescence imaging should be considered as an additional imaging technique to intraoperative ultrasound during surgery for hepatic metastasis.
• Near-infrared fluorescence imaging can aid surgeons in the identification of preoperatively identified liver metastases from primary GIST.
• For the detection of additional superficially located liver metastases from primary GIST near-infrared fluorescence imaging can be used.
• Real-time evaluation of resection margins with NIRF imaging may lead to a lower number of R1 resections.

## 1 Introduction

Gastrointestinal stromal tumors (GIST) are the most prevalent mesenchymal tumors of the gastrointestinal tract, with an incidence of 0.78 per 100.000 people per year [1]. GIST are most commonly located in the stomach (40–60%) and the proximal intestine (20–40%). Distant metastases are mainly found in the liver and peritoneum, while lymph node metastases are rare [2]. Overall 5-year GIST-specific survival rates of 79% have been described, decreasing to 60% when having metachronous or synchronous distant metastases [1]. Without extrahepatic disease, surgery is the treatment of choice for hepatic metastases of GIST.

Indocyanine green (ICG) has been used as fluorescent dye for determining liver function, liver anatomy and for guiding liver resections, with increased utilization over the past decade. ICG binds to plasma proteins and is rapidly taken up by hepatocytes before its excretion into the biliary system. Near-infrared fluorescence (NIRF) imaging shows a variability in distribution of ICG between different tumor types [3]. Previously, we reported that intravenous injection of 10 mg (5 mg/mL) of ICG 24 h prior to surgery leads to a typical rim-shaped fluorescence signal surrounding colorectal liver metastases (CRLM) [4,5]. Extensive research has been conducted to prove the efficacy and feasibility of NIRF-imaging with ICG in the treatment of CRLM. A multicenter retrospective analysis of NIRF-guided resections of CRLM showed that in significantly more patients additional lesions were found intraoperatively using both ultrasound and NIRF imaging compared to ultrasound only [6]. In addition, Tummers et al. reported a case series in patients with liver metastasis originating from uveal melanoma [7]. NIRF-guided surgery with ICG is standard-of-care for patients with all liver metastases in our institution. Although no clinical studies have been published focusing on GIST metastases, we hypothesized that these patients could also benefit from NIRF-imaging using ICG.

Here we report on two cases of open liver resection and radiofrequency ablation (RFA) of hepatic GIST metastases. Informed consent for publication was obtained from both patients and this work is reported according to the SCARE criteria [8,9].

## 2 Case presentation

### 2.1 NIRF Imaging procedure

According to the hospital’s standard-of-care protocol for secondary liver tumors, both patients were admitted to the hospital and were intravenously injected with 10 mg (5 mg/mL) ICG (Verdye® Diagnostic Green GmbH, Asscheim, Germany) 24 h prior to surgery. During the surgical procedure, after access to the abdominal cavity was acquired, a visual inspection of the abdomen was performed. After the liver was fully mobilized, intraoperative open-field NIRF-imaging of the liver was performed, prior to intraoperative ultrasonography (IOUS), using the Quest Spectrum system (Quest, Middenmeer, The Netherlands) to localize the metastases, and identify possible additional lesions. This was followed by resection and/or RFA of the lesions. The surgical procedures were performed by HH and JM, having 12 years of experience with NIRF-guided liver surgery. After surgery the resected specimens were assessed by a pathologist, and closed-field NIRF-imaging was performed with the Pearl Trilogy Imaging System (LI−COR Nebraska, USA) for further inspection of the tumor bread loaves.

### 2.2 Case A

A 69-year-old female was seen at the outpatient clinic for follow-up after resection of a GIST of the stomach (mitotic count 10/50 HPF). Other medical history was notable for laparoscopic cholecystectomy, pulmonary embolism, cerebral infarction, and pyramidal tract syndrome. The CT-scan revealed a suspicious superficial hypodense lesion of 34 × 44 mm in segment VIII and a 4 × 5 mm suspicious lesion in segment VI. In segments V and VIII two preexistent calcifications were found, thought to originate from intraoperative spillage of bile stones during previous cholecystectomy. There was no sign of recurrence of the primary tumor, and no lymph nodes or other distant metastases were found. The primary tumor was a ‘wild-type’ GIST and therefore neoadjuvant chemotherapy was not indicated [10]. She was planned for open resection of the metastasis in segment VIII and RFA for the lesion in segment VI.

**Fig. 1:**
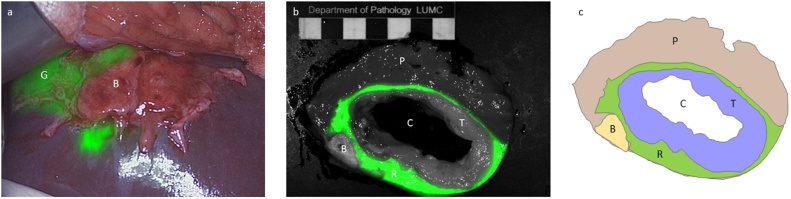
**Fluorescent images of segment VIII in patient A.** a: showing ICG retention around the GIST metastases (G), notice the absence of fluorescence around the calcification (B); b: example of a bread loaf image with closed-field back-table imaging; and c: schematic segmentation of the bread loaf. Bread loaf of a metastasis showing liver parenchyma (P), the cavum (C), a ring-shaped tumor (T), with clear rim-shaped ICG enhancement pattern (R) and a calcified nodule (B).

**Fig. 2:**
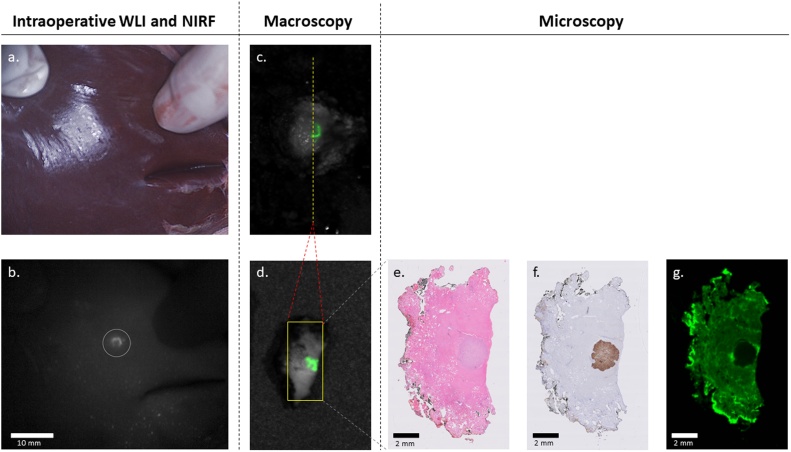
**Correlation between *in vivo* and *ex vivo* NIR fluorescence imaging and microscopy in patient A.** a) White light image of liver segment V with no identifiable tumor; b) NIR fluorescence showing a typical rim-shaped enhancement pattern (circle) suspect for metastatic disease; c) back-table closed field NIR fluorescence overlay image of resected specimen; d) back-table closed field NIR fluorescence overlay image of bread loaf of c); e-g) microscopy images of 4 μm thick slide of d) – H&E staining, DOG1-staining and fluorescence microscopy, respectively.

Intraoperative NIRF-imaging clearly revealed the lesion in segment VIII (Fig. 1). The lesion in segment VI was not visualized using NIRF-imaging, due to its deeper location in the liver parenchyma. In addition, one suspected lesion in segment V was found. This lesion showed a specific rim feature (Fig. 2a, b), similar to what is seen around other secondary liver tumors. IOUS was able to detect the two known tumors in segments VIII and VI, and the previously described calcifications. The newly found lesion was not observed on IOUS. First, ultrasound-guided RFA was performed of the lesion in segment VI, followed by resection of the newly found lesion in segment V and the metastasis in segment VIII.

After completion of the resection, all resected specimens were cut into 5 mm bread loaves by the pathologist, followed by consecutive NIRF bread loaf imaging. The bread loaves were further processed for histopathological analysis and to determine resection margins. Immunohistochemical staining with Discovered on GIST 1 (DOG1), a gene encoding anoctamin 1 – a chloride channel protein [11] – was performed and confirmed tumor localization in both resected specimens, proving the liver metastases originated from a GIST (Fig. 2e–g). During admission the patient developed pulmonary embolisms in both lungs for which she was treated with a nadroparin. After nine days the patient was discharged from the hospital.

Five months post-surgery a new hypodense lesion was found on CT during regular follow-up. After a multidisciplinary team meeting the patient was scheduled for percutaneous RFA. The first post-RFA scan showed no residual disease after RFA. However, two new liver lesions were found, for which the patient was referred to the oncologist for systemic therapy.

### 2.3 Case B

The second case represents an 82-year-old male with a solitary suspicious hypodense lesion in segment III of 21 mm, discovered on routine CT-scan during follow-up after previous partial gastrectomy for GIST (mitotic count 2/50 HPF). Previous medical history reported tuberculosis, acute myocardial infarctions, and a GIST in the stomach. After a multidisciplinary team meeting, the patient was planned for percutaneous, CT-guided RFA. However, the metastasis was located in close relation to the intestine and the RFA procedure was aborted. Instead a biopsy of the lesion was taken, confirming a metastasis from a primary GIST. The patient was planned for open resection of the metastasis. Prior to surgery, another CT-scan was performed, revealing a second 4 mm suspicious lesion in segment VIII. Because of its deeper location, the new lesion was considered to be better suitable for RFA.

**Fig. 3:**
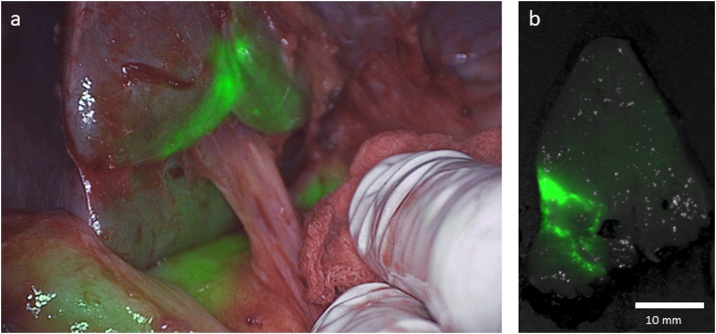
**Correlation between *in vivo* and *ex vivo* NIR fluorescence imaging in patient B.** a) *In vivo* image showing diffuse ICG retention around the tumor; b) corresponding bread loaf image with the closed-field fluorescence imaging system.

After mobilizing the liver, NIRF-imaging revealed the lesion in segment III (Fig. 3a). The second lesion, in segment VIII, was not found with NIRF-imaging due to its deeper location (24 mm subcapsular) in the liver. Both lesions were identified with IOUS. No additional lesions were discovered. Surgical resection was performed for the lesion in segment III followed by RFA for the lesion in segment VIII.

Closed-field NIRF-imaging confirmed the intraoperative findings and showed tumor-free resection margins on the bread loaves (Fig. 3b). Immunohistochemical staining confirmed a metastatic tumor of 8 mm originating from GIST. Microscopic evaluation by the pathologist of the resected specimen also confirmed tumor-free resection margins of 5 mm. After six days, the patient was discharged from the hospital and was seen at the outpatient clinic two weeks after discharge without any complications. Because of his age, the patient decided to refuse any further follow-up.

## 3 Discussion

To the best of our knowledge, this is the first case report solely describing the use of NIRF-imaging with indocyanine green in patients with hepatic metastases from GIST. Positive resection margins and missed tumors following surgery are directly related to a decrease in disease free survival and 5-year survival rates in CRLM [6,12], we emphasized the importance of precision surgery of liver resections in patients with hepatic metastases from GIST.

We described the use of ICG in hepatic metastases from GIST in two patients in our institution. Intraoperative NIRF-imaging showed to be feasible and effective in both localizing preoperatively found superficial metastases as well as finding additional superficial lesions. ICG is currently used as standard-of-care as intraoperative fluorescent contrast agent in our center for liver metastases.

NIRF light penetrates tissue up to 8 mm, allowing the surgeon to identify subcapsular lesions invisible for the naked eye. However, the limited penetration depth remains an important limitation for deeper located lesions. Therefore, IOUS is still required for the identification of deeper (>8 mm) lesions. NIRF-imaging with ICG has proven to be useful for surgical planning and evaluation of resection margins [6]. Imaging systems with higher specificities, as well as new tumor-specific fluorophores, could potentially increase penetration depths and lead to lower false-positive rates.

NIRF-imaging has shown to be an effective imaging technique in the surgical treatment of CRLM, and now in hepatic GIST metastases as well.

## 4 Conclusion

NIRF-imaging with ICG is useful for identification of preoperatively discovered lesions, surgical resection planning and margin evaluation, and for detection of additional hepatic GIST metastases for capsular lesions and subcapsular lesions up to 8 mm below the liver surface. Because of the limited penetration depth of NIRF-imaging should be used as an additional imaging technique next to IOUS. Development of tumor-specific fluorophores for primary GIST and metastases can aid in more accurate detection of these tumors and may lead to lower false-positive rates.

## Declaration of Competing Interest

The authors report no declarations of interest.

## Funding

No funding was received.

## Ethical approval

According to the local medical ethics committee (METC-LDD) no formal ethical approval was required.

## Consent

Written informed consent was obtained from the patient for publication of this case report and accompanying images. A copy of the written consent is available for review by the Editor-in-Chief of this journal on request.

## Registration of research studies

Not applicable

## Guarantor

Okker D. Bijlstra and Alexander L. Vahrmeijer.

## Provenance and peer review

Not commissioned, externally peer-reviewed.

## CRediT authorship contribution statement

**O.D. Bijlstra:** Conceptualization, Methodology, Validation, Formal analysis, Writing - original draft. **F.B. Achterberg:** Methodology, Writing - original draft. **Q.R.J.G. Tummers:** Writing - review & editing. **J.S.D. Mieog:** Writing - review & editing. **H.H. Hartgrink:** Writing - review & editing. **A.L. Vahrmeijer:** Writing - review & editing, Supervision.
